# The combined analgesic effect of pregabalin and morphine in the treatment of pancreatic cancer pain, a retrospective study

**DOI:** 10.1002/cam4.3779

**Published:** 2021-02-16

**Authors:** Junzhu Dai, Lei Teng, Liuyuan Zhao, Huichao Zou

**Affiliations:** ^1^ Department of Pain Medicine Harbin Medical University Cancer Hospital Harbin China

**Keywords:** morphine, pancreatic cancer pain, pregabalin

## Abstract

**Background:**

Pregabalin is commonly used to relieve neuropathic pain. However, data are lacking on its efficacy for the treatment of chronic cancer pain. The purpose of this study was to determine the analgesic efficacy of pregabalin combined with morphine in the management of pancreatic cancer pain.

**Methods:**

This study reviewed patients who were prescribed morphine and 150 mg/d pregabalin between 1 January 2017 and 10 November 2018 in our institute. The primary outcomes of this study were the average pain score and dose of morphine. Secondary outcomes included characters of breakthrough cancer pain, functional interference related to pain, anxiety/depression status, and incidence of treatment‐related adverse events during the study.

**Results:**

A total of 240 patients with pain related to pancreatic cancer were included in the study. The results showed that patients of both combination therapy group (pregabalin+morphine) and monotherapy group (morphine) achieved similar analgesic efficacy, demonstrated by NRS (2.4 ± 0.9 vs. 2.6 ± 0.9; combination vs. monotherapy) at the end of the study. Mean daily dose of morphine used in the combination group was significant lower compared to monotherapy group (39.5 ± 16.0 mg vs. 61.5 ± 19.3 mg, net difference 23.5, 95% CI: 18.4–28.6, *p* <  0.001). The change of functional interference score related to pain was significantly different between combination and monotherapy group (12.0 ± 0.4 vs. 9.8 ± 4.9; net difference, 2.3; 95% CI: 1.1–3.3; *p* < 0.001). Patients in combination therapy group had experienced shorter duration of breakthrough cancer pain than those in monotherapy group (X^2^ *p* < 0.001, Cramer's V:0.36). The incidence of somnolence, dizziness, and cognitive dysfunction were significantly higher in the combination group compared to monotherapy group. No serious treatment‐related side effects were observed.

**Conclusions:**

The findings of this study supported the use of pregabalin with morphine to relieve pain in patients of pancreatic cancer.

## Graphical abstract

Cancer related pain is difficult to manage and caused significant lower quality of life in pancreatic cancer patients. Pregabalin has been widely used for the management of neuropathic pain including cancer related neuropathic pain. This study demonstrates that pregabalin and morphine combination achieved satisfactory analgesic effect with significant lower dose of morphine compared to morphine treatment alone. The finding of this study provides evidence for using pregabalin with morphine in the palliative treatment of pancreatic cancer pain.

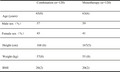

## 1 INTRODUCTION

Pain is one of the most common complications in patients with cancer. The prevalence of cancer pain is approximately 50% at the early stage and is 75% at advanced stage.^1^ Pain caused by pancreatic cancer can be extremely difficult to manage.^2^ Opioid analgesics are the gold standard medications for pancreatic cancer pain.^3^ Continuous escalation of opioid dosage is needed to maintain analgesic efficacy for pancreatic cancer pain.^4^

To reduce the dose and minimize side effects of opioid medications, multimodal analgesia combining various analgesics with different mechanisms is used in chronic cancer pain management. Adjuvant drugs such as hormone steroids, nonsteroidal anti‐inflammatory drugs (NSAIDs), antidepressants, and anticonvulsants are commonly applied with opioid medications in the treatment of chronic cancer pain.^5^ Pregabalin is an anticonvulsant which has been approved for the treatment of neuropathic pain.^6^ Previous studies have found that perioperative use of pregabalin achieved lower pain scores with less dosage of opioid medications.^7^ In our institute, pregabalin has been routinely used in patients with chronic neuropathic pain. It is necessary to explore whether the use of pregabalin is beneficial for patients with cancer pain. To investigate the efficacy and tolerability of pregabalin for patients with pancreatic cancer pain, we conducted this retrospective study.

## 2 METHODS

### 2.1 Participants

The study was conducted at the Department of Pain Medicine in Harbin Medical University Cancer Hospital between 1 January 2017 and 16 November 2018. The protocol was approved by the Institutional Review Board of Harbin Medical University Cancer Hospital. We identified patients with a diagnosis of pancreatic cancer in our hospital electronic information system. The data were collected and reviewed by research assistants and student. During this period, a total of 368 patients of pancreatic cancer were admitted for analgesic treatment in our department. Patients of pancreatic cancer were included in this study if they had pain symptom related to local pancreatic cancer lesion. Patients with neuropathic pain caused by chemotherapy were not included. Patients with other conditions that might induce pain such as biliary obstruction, cholangitis, or infection, change of analgesic therapy with the potential to influence pain such as addition of anticonvulsants, antidepressants, or NSAIDs during the study were not included. Patients were excluded if they lacked follow‐up data. After selection, 240 patients were included in the study. Patients were divided into two groups according to analgesic type: monotherapy (morphine only, n = 120) and combination therapy (morphine+pregabalin, n = 120).

### 2.2 Study medications and pain analgesic program

Two groups of patients were reviewed based on the medications used. Patients in the combination group received Morphine Hydrochloride Sustained‐Release Tablet (Southwest Pharmaceutical) orally every 12 h with pregabalin Capsules (Lyrica; Pfizer) 75 mg twice a day. Patients in the monotherapy group received Morphine Hydrochloride Sustained‐Release Tablets orally every 12 h alone. Immediate release Morphine Hydrochloride Tablets (Northeast Pharmaceutical) were provided for the rescue management of breakthrough pain. The dosage of Morphine Hydrochloride Sustained‐Release Tablets was determined by titration to control Numerical Rating Scale (NRS) score less than or equal to 3.^8^ If the NRS score was more than 3, or there were more than 3 episodes of breakthrough pain in 24 h, then, the daily dosage of Morphine Hydrochloride Sustained‐Release Tablets was titrated again.

### 2.3 Efficacy and safety outcomes

The primary outcome were pain reduction on the basis of NRS score and dosage of morphine by week 4. NRS score was assessed on a 0–10 numerical scale with 0 corresponding to “no pain” and 10 to “the worst possible pain.” Mean dosages of daily morphine were recorded. Secondary outcomes included assessment of functional interference related to pain (Brief Pain Inventory, BPI),^9^ characters of breakthrough pain episode (number, severity, and duration), and mood of patients (Hospital Anxiety and Depression Scale, HADS).^10^ The Brief Pain Inventory assesses the severity of pain and its impact on patients’ functioning such as general activity, mood, walking ability, normal work, relations with other people, sleep, and enjoyment of life. Higher BPI scores represent a greater level of behavior problems caused by pain. The Hospital Anxiety and Depression Scale is a questionnaire to detect states of anxiety and depression of the patients. The HADS questionnaire has seven items each for depression and anxiety subscales. Scoring for each item ranges from zero to three, with three denoting highest anxiety or depression level. A total subscale score of >8 points out of a possible 21 denotes considerable symptoms of anxiety or depression. All patients admitted in our department were routinely evaluated using NRS, BPI, and HADS. Adverse events associated with treatment including constipation, dizziness, nausea, vomiting, and cognitive disturbance were reviewed.

### 2.4 Statistical analysis

All the statistical analyses were calculated using SAS, 9.2 software (SAS Institute). Patient demographics and characteristics were expressed as mean (standard deviation, SD), number and percentage. We considered *p* < 0.05 statistically significant. Analysis of covariance and a repeated measures model were used to analyze NRS, dose of morphine, BPI score, and HADS score. Characters of breakthrough cancer pain and adverse events outcomes were compared between groups using x^2^ test and Cramer's V.^11^ Cramer's V was used to measure the strength of association (weak: >0.05; moderate: >0.10; strong: >0.15; and very strong: >0.25).

## 3 RESULTS

The patient disposition is shown in Table 1. A total of 240 patients with pain due to pancreatic cancer were reviewed including 120 patients in the combination group and 120 patients in the monotherapy group. No significant differences in age, weight, and height were observed between two groups.

**TABLE 1:** Patient demographic

	Combination(n = 120)	Monotherapy(n = 120)
Age (years)	65(8)	63(6)
Male sex (%)	57	59
Female sex (%)	43	41
Height (cm)	168(6)	167(5)
Weight (kg)	57(8)	55(8)
BMI	20(2)	20(2)

Data given as mean (SD).

Abbreviation: BMI, body mass index.

Table 2 showed that combination therapy resulted similar pain relief compared to monotherapy. The mean baseline NRS score was 6.4 ± 1.5 in the combination group and 6.3 ± 1.3 in the monotherapy group (*p* = 0.27). After 4 weeks, the mean NRS score had decreased to 2.4 ± 0.9 in the combination group and 2.6 ± 0.9 in the monotherapy group (*p* = 0.184).

**TABLE 2:** Primary outcome

Outcome	Combination(n = 120)			Monotherapy(n = 120)			Net Difference between groups in change (95% CI)	*p*
	Baseline	4 weeks	Change from baseline	Baseline	4 weeks	Change from baseline		
NRS	6.4(1.5)	2.4(0.9)	−4.0(1.8)	6.3(1.3)	2.6(0.9)	−3.7(1.6)	0.3(−0.1–0.7)	0.184

Data given as mean (SD). NRS Score range: 0–10. Higher scores indicate worse pain. Net difference between groups was calculated as change in combination group minus change in monotherapy group. Analysis of covariance and a repeated measures model were used to analyze NRS.

Abbreviations: CI, confidence interval; NRS: numeric rating scale.

Table 3 listed the dosage of morphine used in both groups. The mean daily dose of morphine at baseline was 18.8 ± 12.5 mg in the combination group, and 17.3 ± 12.2 mg in the monotherapy group. After 4 weeks, mean daily dose of morphine was (39.5 ± 16.0 mg), which was significant lower in combination group compared to 61.5 ± 19.3 mg in monotherapy, yielding between group difference of 23.5 mg (95% CI, 18.4–28.6; *p* < 0.001).

**TABLE 3:** Dose of analgesic drug

Outcome	Combination(n = 120)			Monotherapy(n = 120)			Net Difference between groups in change (95% CI)	*p*
	Baseline	4 weeks	Change from baseline	Baseline	4 weeks	Change from baseline		
Morphine dose (mg)	18.8(12.5)	39.5(16.0)	20.8(16.7)	17.3(12.2)	61.5(19.3)	44.3(21.6)	−23.5(18.4–28.6)	0.000

Net difference between groups was calculated as change in combination group minus change in monotherapy group. Analysis of covariance and a repeated measures model were used to analyze morphine dose.

Data given as mean (SD).

Abbreviations: CI, confidence interval.

Table 4 listed the characteristics of breakthrough cancer pain. There was no difference in numbers and severity of breakthrough pain between two groups. Patients received combination therapy had a significant decrease in the duration of breakthrough pain compared to monotherapy (X^2^ *p* < 0.001, Cramer's V:0.36).

**TABLE 4:** Breakthrough pain characteristics between groups

Feature	Combination(n = 120)		Monotherapy(n = 120)		P(Cramer's V)
	No.	%	No.	%	
No. of episodes					
0–3	88	73.3	88	73.3	0.574(0.07)
4–6	22	18.3	27	22.5	
>7	10	8.3	15	12.5	
Severity (NRS)					
0–3	64	53.3	57	47.5	0.664(0.06)
4–6	42	35.0	47	39.2	
>7	14	11.7	16	13.3	
Duration of episode (minutes)					
0–10	62	51.7	26	21.7	0.000(0.36)
10–30	25	20.8	24	20.0	
30–60	15	12.5	45	37.5	
>60	18	15.0	25	20.8	

Abbreviations: NRS, numeric rating scale.

Outcomes between groups were compared using x^2^ test. Cramer's V was used to measure the strength of association (weak: >0.05; moderate: >0.10; strong: >0.15; and very strong: >0.25).

Table 5 detailed the BPI scores and the HADS scores. Both combination and monotherapy group had achieved similar analgesic effect in terms of BPI pain severity (*p* = 0.813). The reduction of mean BPI interference total score was significantly greater in combination group than monotherapy group (12.0 vs. 9.8; net difference, 2.3; 95% CI: 1.1–3.4; *p* < 0.001). There was significant difference in change of HADS score between combination group and monotherapy group (4.3 vs. 2.7; net difference, 1.7; 95% CI: 0.6–2.8; *p* = 0.004).

**TABLE 5:** BPI and HADS scores between groups

Outcome	Combination(n = 120)			Monotherapy(n = 120)			Net Difference Between Groups in Change (95% CI)	P
	Baseline	4 weeks	Change from Baseline	Baseline	Weeks	Change from Baseline		
BPI severity	6.2(1.4)	2.5(1.1)	−3.6(1.7)	6.1(1.7)	2.5(1.0)	−3.6(2.1)	0.1(−0.4–0.5)	0.813
BPI Interference subscale								
General activity	5.5(1.5)	3.3(1.2)	−2.2(2.0)	6.0(1.3)	3.2(1.1)	−2.8(1.8)	−0.6(−1.1–−0.1)	0.015
Mood	5.2(1.3)	2.8(0.9)	−2.4(1.6)	5.8(1.4)	3.5(1.2)	−2.4(1.6)	0.1(−0.4–0.5)	0.843
Walking	3.3(1.5)	2.9(1.0)	−0.4(1.6)	2.6(1.3)	2.0(1.1)	−0.6(1.6)	−0.2(−0.6–0.2)	0.319
Normal work	5.8(1.3)	4.7(1.1)	−1.1(1.6)	3.8(1.3)	2.1(1.0)	−1.6(1.5)	−0.5(−0.9–−0.1)	0.010
Social relation	3.7(1.0)	3.2(1.1)	−0.5(1.5)	2.4(1.3)	2.1(1.1)	−0.3(1.7)	0.2(−0.2–0.6)	0.364
Sleep	6.0(1.4)	2.8(0.9)	−3.2(1.7)	5.4(1.6)	4.5(1.2)	−0.9(2.1)	2.3(1.9–2.8)	0.000
Life enjoyment	5.8(1.6)	3.6(1.3)	−2.1(2.1)	5.6(1.4)	4.4(1.5)	−1.2(1.9)	1.0(0.5–1.5)	0.000
Total	35.3(3.5)	23.2(2.8)	−12.0(4.4)	31.5(3.8)	21.7(3.0)	−9.8(4.9)	2.3(1.1–3.4)	0.000
HADS	25.7(5.1)	21.4(4.9)	−4.3(4.9)	23.7(5.1)	21.1(4.3)	−2.7(5.7)	1.7(0.6–2.8)	0.004

Analysis of covariance and repeated measure model were used to analyze the outcome.

Abbreviations: BPI, Brief Pain Inventory; CI, confidence interval; HADS, Hospital Anxiety and Depression Scale.

Table 6 listed the common adverse events (AEs) related to analgesic treatment between two groups. Patients in the combination therapy group had significant higher incidence of AEs (X^2^ *p* = 0.001; Cramer's V:0.22). Patients in the combination group experienced more cognitive disturbance (X^2^ *p* = 0.002; Cramer's V: 0.20), somnolence (X^2^ *p* = 0.001; Cramer's V:0.22), and dizziness (X^2^ *p* < 0.001; Cramer's V:0.25) than the patients in the monotherapy group. No serious adverse events including angioedema, seizure, decreased level of consciousness, respiratory depression, or increased suicide risk were reported.

**TABLE 6:** Adverse events related to the treatment

Adverse events	Combination(n = 120)		Mono(n = 120)		P(Cramer's V)
	No.	%	No.	%	
Total No. AEs	88	73.33	62	51.67	0.001(0.22)
Patients with AEs	46	38.33	33	27.50	0.074(0.12)
Serious AEs	0	0.00	0	0.00	‐
Dizziness	35	29.17	11	9.17	0.000(0.25)
Somnolence	28	23.33	9	7.50	0.001(0.22)
Headache	9	7.50	8	6.67	0.801(0.02)
Vomiting	16	13.33	23	19.17	0.221(10.08)
Cognitive dysfunction	26	21.67	9	7.50	0.002(0.20)
Increased pain*	2	1.67	3	2.50	1.000(−0.03)
Nausea	22	18.33	28	23.33	0.340(−0.06)

Outcomes between groups were compared using x^2^ test. Cramer's V was used to measure the strength of association (weak: >0.05; moderate: >0.10; strong: >0.15; and very strong: >0.25).

Abbreviations: AEs, adverse events.

## 4 DISCUSSION

Our study is the first to demonstrate that coadministration of pregabalin with morphine achieved satisfactory analgesic efficacy in patients of pancreatic cancer with reduced dose of morphine. This finding has considerable implications for clinical practice because pregabalin has been increasingly used in the setting of cancer‐related pain. However, the use of pregabalin in patients with cancer pain are usually from anecdotal experience with limited or no evidence from clinical trials.

The mechanism of pancreatic cancer pain is complex. Inflammation in tumor microenvironment, direct infiltration of sensory neurons by tumor, and neuroplastic changes in the sensory neurons all contribute the background pain and unpredicted breakthrough pain episodes in patients of pancreatic cancer.^12^, ^13^ The management of pancreatic cancer pain is based on the WHO pain treatment ladder.^14^ Morphine is cornerstone in the management of pancreatic cancer pain and can provide effective analgesia in some patients with pancreatic cancer pain. However, higher dose of morphine may have considerable side effects and may accompany opioid tolerance which need even higher dose of opioid drug.^15^ Besides constipation, nausea, vomiting, and other common side effects, opioid medications may be associated with worse outcome of cancer. It has been shown that morphine has effects on tumor angiogenesis, cell proliferation, tumor progression, immune function, and metastatic potential.^16^, ^17^, ^18^, ^19^ Thus, management of pancreatic cancer pain need include adjuvant drugs that work through different mechanisms to achieve additive or synergistic analgesic effect with reduced use of opioid drugs. The mechanism of action of pregabalin is reduction of the release of excitatory neurotransmitters by binding the alpha‐2‐delta (α2δ) subunits of the voltage activated calcium channels.^20^ The binding of pregabalin with α2δ subunits binds of gabapentinoids inhibits cellular calcium influx and attenuates neurotransmission which accounts for the action of pregabalin in pain and seizure management. The different antinociceptive mechanism of pregabalin from morphine implicates the possible combinational use of both drugs for chronic pain management.

Several studies have shown the analgesic efficacy of using pregabalin in perioperative setting.^21^, ^22^ However, the role of pregabalin in cancer‐induced pain is controversial. In a study of patients with cancer‐induced bone pain, pregabalin combining with palliative radiotherapy did not show additional benefit compared to patients receiving radiotherapy alone.^23^ However, in a study of patients with radiotherapy‐related neuropathic pain, pregabalin showed greater pain alleviation, better mood states, and higher quality of life compared with patients treated with placebo.^24^ Despite of the controversial conclusions, pregabalin is often prescribed for cancer‐related pain, especially if there is a neuropathic component to the cancer pain. Our findings from this retrospective study support the use of pregabalin for pancreatic cancer‐related pain.

In this study, both groups achieved satisfactory analgesic effect. The pain intensity reduction compared to baseline in the combination group and monotherapy group was 4.0 and 3.7, respectively. The mean daily dose of morphine in the combination group was 39.5 mg which was significantly lower compared to the 61.50 mg in monotherapy group. Addition of pregabalin resulted similar analgesic effect with 36% reduction of morphine dose in patients with pancreatic cancer pain. The dose change usually starts from 25% to 50% of the baseline when titrating the dose of morphine,^25^, ^26^, ^27^ thus, the 36% reduction of morphine dose is considered significant for clinical practice.

Cancer pain is usually accompanied with psychological distress such as anxiety and depression. In this study, we used HADS to assess the change of anxiety and depression before and after the analgesic treatment. We found that patients in the combination therapy group have better improvement of HADS score than the monotherapy group. The established application of pregabalin for general anxiety may contribute the improved HADS score. We have also found that pregabalin and morphine combination treatment resulted in notable improvement in quality of life versus morphine monotherapy. Patients in the combination group had significant less pain‐related interference with patients’ normal work, sleep, and enjoyment of life. This finding is consistent with previous reports of combination of gabapentinoid with morphine improved quality of life.^27^, ^28^

Patients in the combination group experienced more adverse events than those in the monotherapy group. The incidence of somnolence, dizziness and cognitive dysfunction occurred more frequently in the combination group. The effect of pregabalin in the central nervous system may contribute to the increased incidence of adverse events. However, the side effects related to pregabalin are generally mild and tolerable. No serious side effects such as angioedema, seizure, decreased level of consciousness, respiratory depression, and increased suicide risk severe side effects were observed in the study.

Breakthrough pain caused by pancreatic cancer has a significant impact on the patient's life. The mechanism of breakthrough pain has not been fully understood. Persistent noxious stimuli such as inflammation‐induced peripheral and/or central sensitization may partly contribute to the incidence of breakthrough cancer pain. No change of the number of episodes or intensity of breakthrough pain were found in this study with the coadministration of pregabalin with morphine compared to monotherapy group. However, the duration of breakthrough pain episodes in the combination group was significantly shorter than the monotherapy group.

There are some limitations in this study. This study is a retrospective study and a randomized prospective study is needed to validate our findings. The dose of pregabalin in this study was fixed at 75 mg twice a day. This dose was used in our clinical practice to ensure no serious sedation or respiratory difficulty occurred. A personalized higher dose of pregabalin may achieve better analgesic efficacy. The follow‐up period of this study was 4 weeks. A longer follow‐up period is needed to validate the analgesic efficacy and impact on quality of life of pregabalin in patients with pancreatic cancer.

In conclusion, this retrospective study showed that pregabalin and morphine combination therapy achieved satisfactory analgesic efficacy with lower dosages of morphine in patients with pancreatic cancer pain. Our results supported the use of pregabalin with morphine in patients who have pancreatic cancer‐related pain.

## Conflict of Interest

The authors declare that they have no conflict of interest.

## Ethics Declarations

This study was conducted in accordance with principles for human experimentation as defined in the Declaration of Helsinki and International Conference on Harmonization Good Clinical Practice guidelines, and approved by the Institutional Review Board of Harbin Medical University Cancer Hospital. Informed consent from the patients was waived by the IRB because the nature of this retrospective study was reanalyzing of existing data which does not involve any potential risks and benefits to the patients.

## Data Availability

The data that support the findings of this study are available on request from the corresponding author. The data are not publicly available due to privacy and ethical restrictions.
